# Identification of a Transferable Linear Plasmid Carrying the Macrolide-Clindamycin Resistance Gene *erm*(X) in a Cutibacterium acnes Isolate from a Patient with Acne Vulgaris in Japan

**DOI:** 10.1128/mra.00094-22

**Published:** 2022-04-19

**Authors:** Juri Koizumi, Keisuke Nakase, Hidemasa Nakaminami

**Affiliations:** a Department of Clinical Microbiology, School of Pharmacy, Tokyo University of Pharmacy and Life Sciences, Hachioji, Tokyo, Japan; University of Arizona

## Abstract

Cutibacterium acnes, one of the common skin bacteria, is known to exacerbate acne vulgaris. Macrolide-clindamycin-resistant C. acnes strains have been reported worldwide. In this study, we found a transferable linear plasmid carrying the macrolide-clindamycin resistance gene *erm*(X) in a C. acnes strain isolated from a patient with acne vulgaris.

## ANNOUNCEMENT

Cutibacterium acnes, a Gram-positive anaerobic bacillus, is one of the common skin bacteria and may exacerbate acne vulgaris by abnormally proliferating in closed hair follicles. Recently, the emergence and increase of macrolide-clindamycin-resistant C. acnes strains, owing to inappropriate antimicrobial use for acne treatments, have become a global concern (1–5). Acquisition of *erm*(X) in C. acnes is associated with high-level resistance to macrolides and clindamycin (6, 7). In C. acnes, *erm*(X) is located on the transposable element Tn*5432* and is considered to be transmitted among C. acnes strains by conjugation (6). In this study, we found a transferable linear plasmid carrying *erm*(X) in C. acnes isolates showing high-level resistance to macrolides and clindamycin.

The C. acnes strain TP-CU411 was isolated from a patient with acne vulgaris in Japan on 28 March 2014 (8). To isolate the C. acnes strain, an acne pustule sample was collected with a sterilized swab and was spread on modified Gifu anaerobic medium (GAM) agar (Nissui Pharmaceutical Co., Tokyo, Japan) containing 20 μg/mL furazolidone (FUJIFILM Wako Pure Chemical Corp., Tokyo, Japan) and 5 μg/mL colistin (FUJIFILM Wako Pure Chemical Corp.). A colony was obtained after culturing for 5 days at 35°C under anaerobic conditions; it was collected, identified as C. acnes by multiplex touchdown PCR (9), and conserved as strain TP-CU411 in 15% glycerol. A single colony of strain TP-CU411 was selected and suspended in modified GAM broth. After incubation, genomic DNA was extracted from the bacterial suspension by the following method. The bacterial pellet obtained by centrifugation was resuspended in resuspension buffer (Tris-EDTA [TE] buffer with 10 μg/mL RNase, 10 mg/mL lysozyme, and 5 mg/mL achromopeptidase [each from FUJIFILM Wako Pure Chemical Corp.]). The suspension was incubated at 37°C for 40 min. The reaction solution was processed in phenol-chloroform-isoamyl alcohol (25:24:1) (10). Whole-genome sequencing was performed using an RS II system (Pacific Biosciences [PacBio], CA). The reads were assembled *de novo* with Hierarchical Genome Assembly Process v3 (HGAP3) within the single-molecule real-time (SMRT) Analysis v2.3.0 software. Prediction of open reading frames and gene annotation were conducted using DFAST v1.1.15 software. NCBI BLAST was used for the comparative analysis of amino acids and base sequences. Default parameters were used for all software unless otherwise specified. BLAST Ring Image Generator (BRIG) (http://brig.sourceforge.net) and Easyfig (https://mjsull.github.io/Easyfig) were used for comparative analysis with known linear plasmids (11, 12).

**FIG 1:**
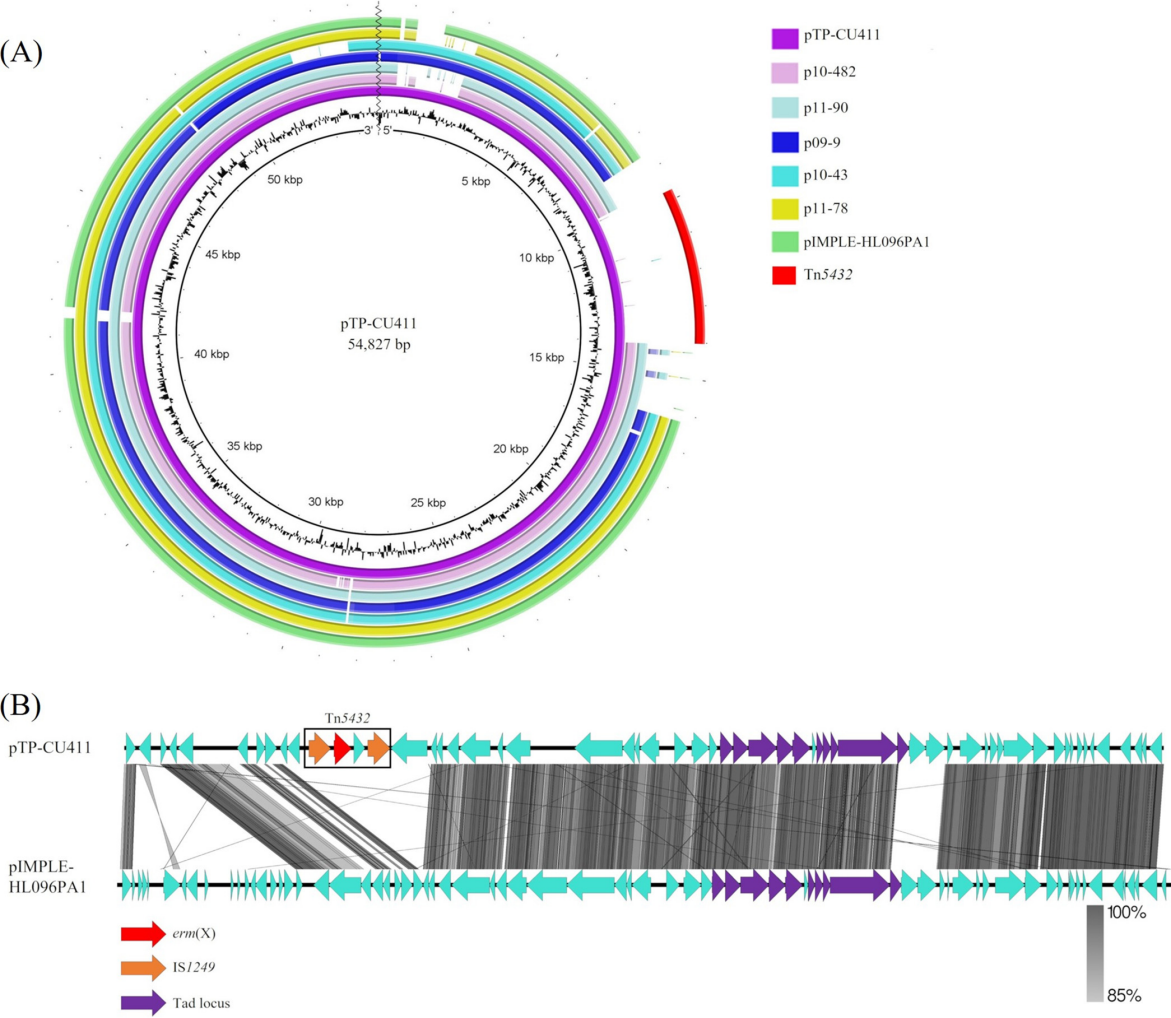
Nucleotide sequence analysis of pTP-CU411. (A) Nucleotide sequence comparison between pTP-CU411 and known linear plasmids in Cutibacterium acnes. pTP-CU411 contained only Tn*5432*, which comprised IS*1249* and *erm*(X) sequences. The sequences of p10-482 (accession number CM008361.1), p11-90 (accession number CM008363.1), p09-9 (accession number NZ_CM008375.1), p10-43 (accession number CM008364.1), p11-78 (accession number CM008362.1), and pIMPLE-HL096PA1 (accession number CP003294) were obtained from GenBank. This figure was generated using BRIG. (B) Insertion of *erm*(X) in pTP-CU411. pIMPLE-HL096PA1 (GenBank accession number CP003294), which showed the greatest nucleotide identity, was compared with pTP-CU411. This figure was generated using Easyfig.

The average read *N*_50_ value was 16,857 bp, with 162,025 subreads. Whole-genome sequencing revealed that C. acnes TP-CU411 had both a chromosome (2,493,567 bp, with a GC content of 60.03%) and a linear plasmid, pTP-CU411 (54,827 bp, with a GC content of 62.15%). Comparative analysis of pTP-CU411 and other known linear plasmids showed a high level of nucleotide identity (97.3% [24,604/25,287 bp]) and 92% query coverage with pIMPLE-HL096PA (13) (Fig. 1A). pTP-CU411 had a 4,455-bp sequence that was not found in any known plasmid. This sequence contained Tn*5432*, as identified in C. acnes strain SP64 (GenBank accession number AF411029.1), with 99.9% nucleotide identity (4,279/4,280 bp) (Fig. 1B). Similar to other known plasmids, pTP-CU411 contained a tight adhesion (*tad*) locus, which is considered to be involved in host adhesion and pathogenicity (14). Linear plasmids have previously been detected in C. acnes clinical isolates (13); however, there are no reports suggesting that a linear plasmid is associated with transmission of antimicrobial resistance. The only known plasmid associated with antimicrobial resistance in C. acnes is pTZC1, which contains *erm*(50) and *tet*(W) (7). It has been suggested that C. acnes linear plasmids are associated with adhesion to host tissues. This study showed that pTP-CU411 is associated with the transmission of antimicrobial resistance.

### Data availability.

The genome sequences of the chromosome and pTP-CU411 of C. acnes strain TP-CU411 (GTC21701 in Gifu University Center for Conservation of Microbial Genetic Resource [GCMR]) were deposited in NCBI GenBank under accession numbers AP025554 and AP025555, respectively. The NCBI Sequence Read Archive (SRA) accession number for the raw reads is DRX335082.
